# Regulation of GM Organisms for Invasive Species Control

**DOI:** 10.3389/fbioe.2019.00454

**Published:** 2020-01-21

**Authors:** Heidi J. Mitchell, Detlef Bartsch

**Affiliations:** ^1^Office of the Gene Technology Regulator, Australian Government Department of Health, Canberra, ACT, Australia; ^2^Federal Office of Consumer Protection and Food Safety, Bundesamt für Verbraucherschutz und Lebensmittelsicherheit, Berlin, Germany

**Keywords:** genome editing, gene drive, environmental risk assessment (ERA), regulation, invasive species

## Abstract

Invasive species can cause significant harm to the environment, agriculture, and human health, but there are often very limited tools available to control their populations. Gene drives (GD) have been proposed as a new tool which could be used to control or eliminate such species. Here, GD describes a variety of molecular biology applications which all enable the introduction of genetic elements at a higher than expected frequency. These elements can change the genotypes in target populations rapidly with consequences either for (intrinsic) fitness or host-parasite interaction, or both. Beneficial applications are foreseen for human and animal health, agriculture, or nature conservation. This rapidly developing technology is likely to have major impacts in the fight against various diseases, pests, and invasive species. The majority of GD applications involve genetic engineering and novel traits. Therefore, applicants and GMO regulators need to interact to achieve the benefits in innovation while cautiously avoiding unacceptable risks. The release into the environment may include transboundary movement and replacement of target populations, with potential impact on human/animal health and the environment. This article summarizes knowledge-based discussions to identify information gaps and analyzes scenarios for responsible introduction of GD organisms into the environment. It aims to connect the latest scientific developments with regulatory approaches and decision-making.

## Introduction

### Impacts of Invasive Species

Invasive species are animals and plants introduced accidentally or deliberately into a natural environment different from the one they originate, with serious negative consequences for their new environment. This definition was taken from a recent JRC report (Tsiamis et al., 2019), which lists such invasive species of EU concern. Invasive species include viruses, microorganisms, fungi, insects, and other invertebrates, feral animals, marine pests, and weedy plants. Invasive species have caused serious adverse effects to human health, agriculture, and the environment. For example, the high rate of extinction of Australian land mammals (>10% of the 273 endemic terrestrial species over the last ~200 years) is likely due primarily to predation by invasive species, particularly feral cats (*Felis catus*) and European red foxes (*Vulpes vulpes*) (Woinarski et al., 2015; Murphy et al., 2019).

For humans, one of the most dangerous effects of invasive species is their direct pathogenic effects or indirect vector activity for disease. In Europe, the Asian tiger mosquito (*Aedes albopictus*) is thought to have been accidently imported from Southern China on recycled tyres and lucky bamboo plants (*Dracaena sanderiana*). It vectors many pathogens, including yellow fever and chikungunya virus (Medlock et al., 2012).

Invasive plants impact the environment (for instance) by outcompeting native plants and reducing agricultural production. But they may also negatively impact human health. Ragweed (*Ambrosia artemisiifolia* L) came to Europe from North America as a contaminant in bird seed. It has spread rapidly and produces highly allergenic pollen that causes hay fever in 4–5% of Europeans (Richter et al., 2013).

These invasive species also cause high economic damages or losses. For example, in Australia the annual cost of pest animals was estimated at $597M in 2013–14 in lost productivity and cost of controls (McLeod, 2016). Similarly, weeds were estimated to cost nearly $5 billion across Australia in 2018. The costs of chemical control in broad acre cropping and lost production costs in the grain, beef and wool industries lead to most of these impacts and damages (McLeod, 2018).

Control or eradication of invasive species once they have established is difficult. Weed and pest control managers need a variety of tools to use in integrated pest management approaches (Messing and Wright, 2006). A number of these biocontrol tools including sterile-release, YY Males, Trojan Female Technique and gene drive were reviewed at the Genetic Biocontrol for Invasive Species Workshop in Tarragona Spain, March 31st, 2019, which was sponsored by the OECD Co-operative Research Programme: Biological Resource Management for Sustainable Agricultural Systems (CRP). The workshop raised awareness of benefits and risks of invasive species control in general, with GD techniques as a case example. The meeting provided the opportunity for an open exchange of views. A summary of the technical and historical developments in this emerging field is presented by Teem et al. (in preparation). The present article highlights regulatory approaches and decision-making for invasive species control including GD.

### Gene Drive (GD) as a Specific Case for Both Introduction and Control of Invasive Species

Gene Drive (GD) describes a variety of molecular biology applications which all enable the introduction of genetic elements that are inherited at frequencies above those predicted by the Mendelian rules that means the transmission of a specific allele to the next generation is greater than the expected 50%. GDs only work in out-crossing sexually reproducing species as they are active in the germline or when the embryo is formed. Gene drives can theoretically spread through the entire population of a species or, depending on the sequence targeted, could be limited to certain areas or populations. However, cage experiments with insects and computer modeling have shown that some gene drives may not spread unchecked through target populations due to the evolution of resistance (KaramiNejadRanjbar et al., 2018) or negative effects on fitness in the target species (de Jong, 2017).

Different types of gene drives occur naturally in a number of species. Meiotic drives have been reported in insects and plants, for example in *Drosophila melanogaster* (McDermott and Noor, 2010) and *Silene latifolia* (Taylor and Ingvarsson, 2003); cytoplasmic incompatibility caused by *Wolbachia* bacteria (Sinkins, 2004) and maternal-effect dominant embryonic arrest (Medea) in flour beetle (*Tribolium castaneum*) (Beeman et al., 1992; Rüdelsheim and Smets, 2018).

An example of how natural gene drives can be utilized to control invasive species is that of *Wolbachia* endosymbiont bacteria (Box 1).

Box 1 **Example of non-GMO gene drive—Regulation of *Wolbachia* containing insects**

Wolbachia are bacteria which infect a wide range of arthropod hosts and manipulate the host reproduction (Sinkins, 2004). They generate a gene drive by causing incompatibility between eggs and sperm or by killing of males. The bacteria are maternally inherited and their manipulation of reproduction favors survival of infected females.

*Wolbachia pipientis* has been introduced into *Aedes aegypti* populations where they greatly reduce the replication of dengue virus and other human pathogens within the infected mosquito (Kambris et al., 2009; Moreira et al., 2009; Bian et al., 2010; Schmidt et al., 2017). As transfer of a whole organism is not considered to lead to a GMO, field trials of this work are regulated by Australian Pesticides and Veterinary Medicines Authority under the *Agricultural and Veterinary Chemicals Code Act 1994* as a veterinary medical product (De Barro et al., 2011).

New molecular techniques enable a previously unachievable spatially and functionally precise modification of the genomes of plants, animals, and microorganisms. These techniques enable a range of changes from site-specific alterations of single nucleotides, to the site-specific insertion of entire genes. Currently, attention is focused on CRISPR Cas9 technology, but many other naturally occurring site specific nucleases can also be used. Engineered gene drives introduce genetic changes with the help of natural components of a gene drive or site specific nucleases. The consequence is a rapid increase of the modified genes in the organism's population.

Internationally, there is rapidly growing research interest in using gene drives for the control of a variety of invasive species. Potential applications include:

- Controlling populations of invasive animals (e.g., exotic rodents), to protect natural environments (Leitschuh et al., 2018; Moro et al., 2018; Harvey-Samuel et al., 2019)
- Controlling invasive plants (weeds) of natural or agricultural environments (Neve, 2018)

Use of GD for control of invasive species, diseases and pests may offer great benefits to society. However, as with any technology for species control, it may also pose risks to wild species or ecosystems. GD raises new challenges for regulation, specifically when the GD involves genetically modified organisms (GMO). GMOs are regulated in most countries, and are also covered by international agreements such as the Cartagena Protocol under the United Nations Convention on Biological Diversity (CBD)^1^.

Regulation of GMOs generally requires an Environmental Risk Assessment to be conducted before a GMO can be released into the environment. The Environmental Risk Assessment starts with the development of risk scenarios—hypotheses of what harm the GMO could cause to people or the environment. The type of data that needs to be collected prior to the release would be informed by these scenarios. Development of the hypotheses generally requires input and advice from a range of different scientific disciplines (see section Specific ERA Challenges Associated With Gene Drive Organisms of this paper).

## Key Elements to Regulation

Most regulatory systems aim to protect human and animal health and the environment while at the same time enabling research and development of beneficial products by modern biotechnology. Since there is no activity in life that does not carry some risk, both regulatory precaution and innovation principles need to consider risks caused by taking action or no action. Avoiding innovation, e.g., by overcautious and restrictive GMO regulations, might also increase the risk of biodiversity loss, food insecurity, and socioeconomic disasters in a time where human population growth, biodiversity loss, climate change, and decreasing natural resources are substantially impacting Earth (Bartsch, 2017). In this respect, evidence based decisions support more sustainable solutions. Decision makers must pay thorough attention to factors relating to both the production and use of such evidence (Redford et al., 2019).

Whilst most regulatory systems for GMOs have the same primary goals different regulatory systems in various jurisdictions may incorporate other issues. For example, Directive 2001/18/EC (EC—European Commission, 2009) recognizes the respect for ethical principles in the EU, and Member States may take into consideration ethical aspects when GMOs are deliberately released. Socioeconomic advantages and disadvantages of each category of GMOs authorized are considered in a report to be issued every 3 years by the EU Commission. The decision maker in the EU is—theoretically—not restrained from considering benefits. In Australia, decisions under the *Gene Technology Act 2000* cannot consider ethics and economics. However, the Australian States and Territories can introduce Policy Principles to consider these.

### The Role of Regulation for Innovation

**Figure 1:**
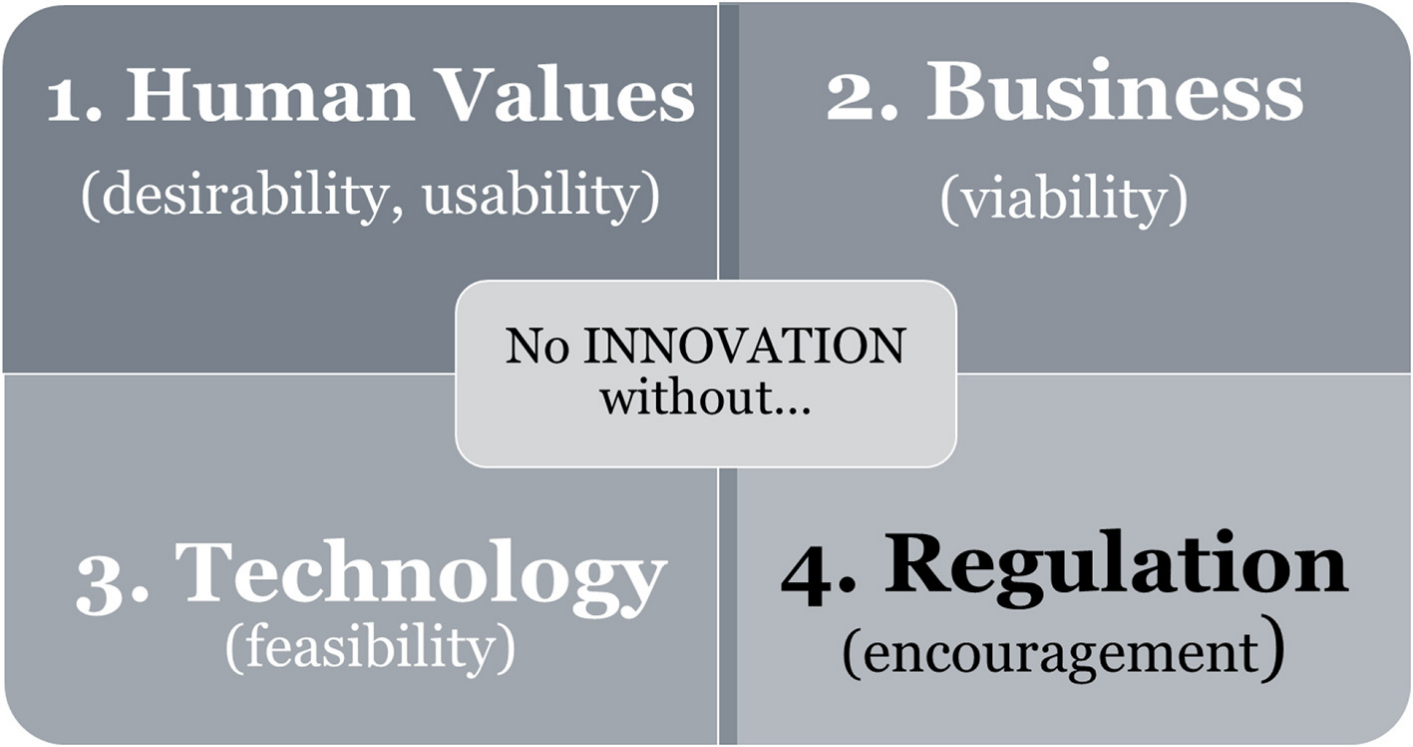
The “four-leaf clover” of innovation. The model is derived from the Theory of Culture Development (altered from Figure 4 of von Thienen et al., 2019). Innovation depends on the requirement of all four leaf branches. Three of them (white letters) evolve directly from [human] culture. Novel needs [including environmental protection goals] based on human values (1) which are only viable if [economic] business (2) is possible in combination with the development of novel [technology] designs (3). As an indirect driver, encouragement by regulation (4) completes the successful leaf development.

Regulation is important for framing innovation, since promotions and restrictions are vital factors guiding which products make it through the research and development stage. Political and economic contexts are important factors influencing technological development and the range of economic profit, and societal need determines technological priorities (see Chapter 6.3 of Redford et al., 2019). Innovation is only possible if new ideas match the desirability/usability for human values, viability of business, technological feasibility, and regulatory encouragement (see Figure 1).

The technological feasibility of gene drives has developed very rapidly and research projects have been initiated across vector control and agriculture (National Academies of Sciences Engineering Medicine, 2016). It is too early to determine the success, business viability or public acceptability in many of these areas yet.

Human values find their expression in protection goals when it comes to the assessment of invasive species. Whether control actions are acceptable depends on the trade-off between environmental/health damage caused by the invasive species/pest/disease vs. undesired off-target effects of the controlling technology. The public concerns about GMOs will also influence this debate (Cormick and Mercer, 2017; Delborne et al., 2019; Hartley et al., 2019).

### Environmental Protection Goals

Since supporting human and valued animal health is a universally accepted protection goal, this section will focus on the environment. The goal of environmental protection is avoiding harm and/or remediation of damage. Bartz et al. (2009) defined environmental damage as:

1. “A significant relevant adverse effect on a biotic or abiotic conservation resource
2. that has an impact on conservation
  - values,
  - ecosystem component, or
  - its sustainable use.”

This definition covers the purposes of conservation as defined in the CBD: “to protect conservation resources themselves and in their role as a part of ecological structures and functions and to safeguard their potential sustainable use.”

The definition has three normative conditions, basically due to legal enforcement options: Only

- concrete (measurable) adverse effects,
- adverse effects that lead to a decrease in “the value,” and
- adverse effects that are significant [environmentally relevant] can be damages.

The magnitude of adverse effects caused by invasive species is reviewed and classified in more detail by Blackburn et al. (2014). However, it is important to re-iterate that regulatory decision making should take into account the ecological consequences of applying/not applying control measures (including GD) when it comes to remediation of damage. A well-designed risk assessment helps to manage the tension between a desire for caution regarding the risk of intervention and worry about the risks of non-intervention (Wareham and Nardini, 2015). A historic example of intervention for the beneficial eradication of a disease is malaria in Europe (Box 2).

Box 2 **Comparative assessment for protection goals—Eradication of malaria in Europe:**

Malaria was a widespread disease in several parts of Europe including Germany (Dalitz, 2005). The eradication was achieved with the help of chemical and sanitary measures to kill the mosquitoes which transmitted malaria (De Zulueta, 1998; WHO, 2016). These included drainage of wetlands in the Oderbruch west of Berlin in the eighteenth century and broad-spectrum pesticide sprays in the Italian Po-Valley. It is not known what unintended environmental damages occurred from these interventions.

Malaria could re-establish by re-introduction of mosquitoes via global trade shipments or tourists arriving from infested countries, combined with the possibility of the receiving environment in the EU being permissible due to global climate change conditions (Schröder and Schmidt, 2014). It is conceivable that GD may become an option to target malaria via eradication or substitution of vector insect populations.

The GD organism released for invasive species control is—although “invasive” to some extent by definition—a beneficial species since it mitigates damage to the environment, human economy or human health. However, the beneficial organism should not turn into an undesired invasive species.

### International Legal Frameworks

International instruments provide valuable frameworks for the regulation of GD (Table 1).

**Table 1:** International legal frameworks (adapted with permission from Redford et al., 2019).

**Instrument**	**Description**	**Relevance for gene drive**
Convention on Biological Diversity (CBD) Adopted: 1992, Entered into force: 1993 Parties: 196	Global legal framework addressing conservation, sustainable use and sharing of benefits of biodiversity	Creates obligations for each Party to manage risks associated with living modified organisms that could have a negative impact on biological diversity [art. 8(g)] and framework for access and benefit sharing relating to genetic resources (art. 15).
Cartagena Protocol on Biosafety to the Convention on Biological Diversity (Cartagena Protocol) Adopted: 2000, Entered into force: 2003 Parties: 171	Protocol to CBD intended to ensure the “safe transfer, handling and use of living modified organisms resulting from modern biotechnology that may have adverse effects on biological diversity…” (art. 1)	Requires sharing of risk related information between exporting and importing Parties and provides guidelines on methodology for environmental risk assessments and considerations in decision-making.
Nagoya-Kuala Lumpur Supplementary Protocol on Liability and Redress to the Cartagena Protocol on Biosafety (Supplementary Protocol) Adopted: 2010, Entered into force: 2018 Parties: 42	Supplementary Protocol to Cartagena Protocol intended to provide rules and procedures for liability and redress relating to living modified organisms	Provides for national frameworks requiring response measures and assigning civil liability in event of damage resulting from living modified organisms which find their origin in transboundary movement.
Nagoya Protocol on Access to Genetic Resources and the Fair and Equitable Sharing of Benefits Arising from their Utilization to the Convention on Biological Diversity (Nagoya Protocol) Adopted: 2010, Entered into force/not entered into force	Protocol to CBD providing international framework for access to genetic resources and sharing of benefits arising from their utilization	Applies to genetic resources that serve as source material for synthetic biology research. Creates ABS framework based on traceability and transfer of material.

Since GD applications are intended to release organisms that become established in the environment and may spread across landscapes, countries have a responsibility for transboundary risk assessment and liability of damage caused by such releases. Many—but not all—countries work under the umbrella of the Cartagena Protocol on risk assessment, information exchange, and further harmonized regulation of transboundary movements of GMOs (Tung, 2014). It is likely that regional and bilateral approaches will be established first before harmonization can be expected at higher international levels.

There is an international customary rule that a country must prevent and provide compensation for damage wrongfully caused from its territory to other states (see more details in Redford et al., 2019). The International Law Commission of the United Nations has published draft articles on the responsibility of countries for internationally wrongful acts. These provide an obligation to make reparation for “any damage, whether material, or moral, caused by the internationally wrongful act of a State” (United Nations, 2001). Whether these rules may apply for negative effects caused by GD releases is—as far as the authors know—not completely solved yet.

### The EU Regulatory System

The EU has elaborate guidelines on application of the precautionary principle which include a preliminary evaluation of risks and uncertainties to determine when the principle is triggered (EU, 2000). The precautionary principle has been taken into account in the drafting of the two statutory regimes:

- Contained use (Directive 2009/41/EC for microorganisms EC—European Commission, 2009, in various EU Member State regulations also for other organisms)
- Release into the environment [Directive 2001/18/EC (EC—European Commission, 2001)].

Contained use in the laboratory is the first step in developing safe and sustainable GD applications. There is currently an initiative for EU wide harmonization on risk assessment and authorization for such use (van der Vlugt et al., 2018) since responsibility falls to the authorities of EU Member states.

**Figure 2:**
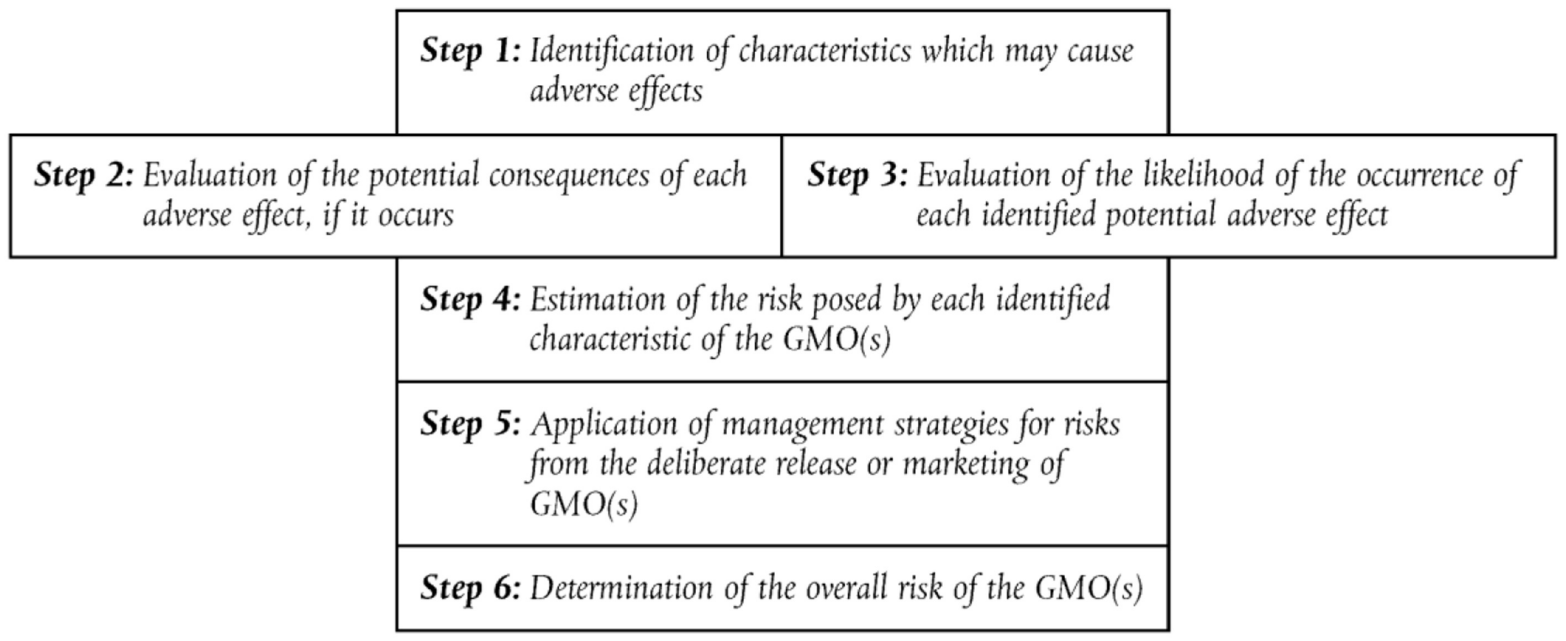
The six steps in the analysis of Environmental Risk Assessment (ERA) in the European Union according to Directive 2001/18/EC (EC—European Commission, 2001).

The Directive 2001/18/EC (EC—European Commission, 2001) sets out a step-by-step approach for introduction of a GMO into the environment, with evaluation of impacts on human health and the environment. Information is required about parental / donor / GM organism, and the receiving environment. Risk assessment follows a case by case and step by step approach (see Figure 2). It is important to identify the characteristics which may cause adverse effects, e.g., effects on the dynamics of populations of species in the receiving environment and the genetic diversity of each of these populations.

#### Regulation of Gene Drives in the EU

**Figure 3:**
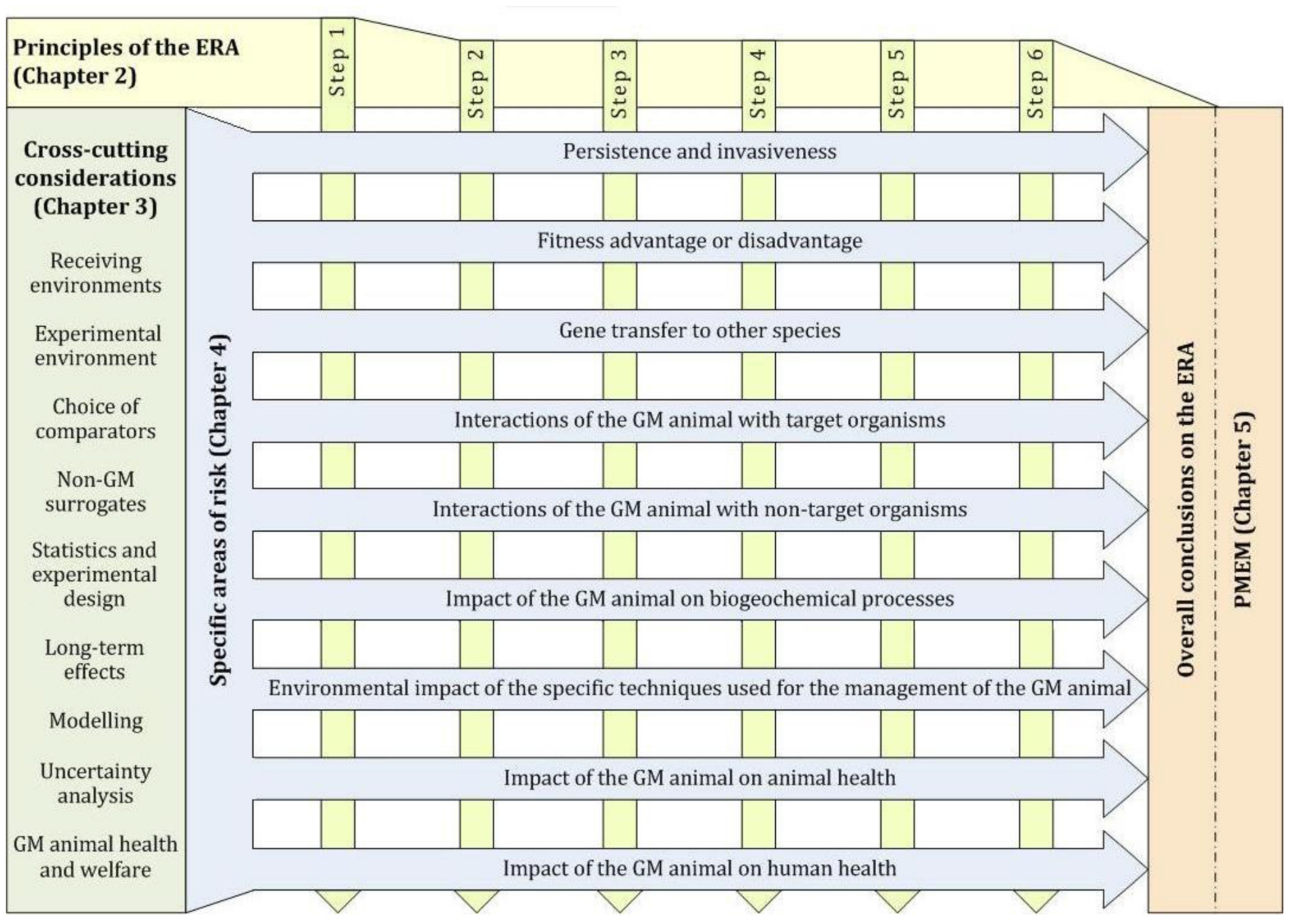
Structure of the EFSA Guidance document on ERA of GM animals, which would apply to GD insects. In late 2018, EFSA received a new mandate from the EU Commission on GMOs engineered with gene drives (gene drive modified organisms) and their implications for RA methodologies. EFSA is requested to identify potential risks in terms of impact on human and animal health and the environment. EFSA is also asked to identify potential novel hazards and to determine whether the existing guidelines are adequate or whether there is a need for updated guidance. EFSA is not requested to develop guidelines for the RA of gene drive modified organisms. Thus, the current guidance on the ERA of GM plants and animals are still valid (EFSA Panel on Genetically Modified Organisms., 2010, 2013).

For GD to control invasive species, the (intended) effect on targeted (invasive) species is not regarded as adverse but beneficial since the invasive species already negatively affects other species in the receiving environment. The environmental risk assessment follows in detail the Guidance Documents published by the European Food Safety Authority (EFSA). Since GD is likely to be first applied in form of GM animals, the structure of the EFSA GD document on ERA of GM animals is shown in Figure 3.

### The Australian Regulatory System

#### Regulation of GMOs

Australia has specific legislation to regulate activities with GMOs to protect people and the environment. The *Gene Technology Act 2000* (Commonwealth of Australia, 2000) and the Gene Technology Regulations 2001 (Commonwealth of Australia, 2001), covers activities with all GMOs, including microorganisms, plants and animals, both in contained facilities and when released into the environment.

The objective of the legislation is to “protect the health and safety of people, and to protect the environment, by identifying risks posed by or as a result of gene technology, and by managing those risks through regulating certain dealings with GMOs.”

Regulation of contained GMOs typically focuses on the suitability of containment. For a GMO intended to be released into the environment, protection of the environment is typically achieved by following a step-wise development process (OECD, 1986): data from initial contained research, overseas release/s or release of a similar GMO inform authorizations for small, short term, confined trials where the GMO is removed from the environment once the trial is finished. Each application for release into the environment requires a case-by-case risk assessment and tailored risk management plans, combined with mandatory consultation requirements, including formal consultation with the Australian Minister for the Environment.

The Risk Analysis Framework (OGTR, 2013) explains the Regulator's methods for risk analysis. It mandates a comparative, problem formulation approach where risk scenarios are used to develop credible causal pathways whereby a GMO may cause harm to people or the environment.

#### Regulation of Gene Drives in Australia

Recent amendments to the GT legislation^2^ provide clarity on the regulatory status of organisms developed using a range of new technologies. Work with organisms containing a functional engineered gene drive will require a specific case-by-case evaluation of the risks and specific risk management of activities with these organisms. This assessment permits information gathering as well as monitoring of the progress of research in this new area. Case-by-case evaluation will take into account any risk-mitigating approaches such as molecular (e.g., split drives, daisy drives, and synthetic targets whereby the gene drive is engineered to prevent uncontrolled spread), environmental or physical containment.

The 2017 legislation review (Commonwealth Department of Health, 2018) observed that “There is an identified need to determine the most appropriate approach for regulating the environmental release of genetically modified gene drive organisms (as well as any additional requirements for contained work).” This may lead to future consideration of whether changes to policy are needed to address issues raised by GD GMOs, particularly in the context of intentional environmental releases.

## Specific ERA Challenges Associated with Gene Drive Organisms

Any ERA should start with the Problem Formulation step in which the ERA scope is determined, including the protection goals and the risk hypotheses. The nature of the GD and its ability to spread could lead to jumping over gradual introduction steps (laboratory—small scale release—large scale release) into the environment. Careful consideration of data gaps related to this “short-cut” is inherently important.

Gene drives can be designed to be self-limiting, whereby the drive will only work for a limited number of generations or is limited spatially. If a GD is designed to be self-limiting, the evaluation of population suppression GDs needs to consider the limited GD persistence in the environment and the required efficacy of the GMO release.

If a GD is designed to be self-sustaining, population suppression GDs need to consider the higher persistence in the environment and the smaller number of required releases of modified organisms. The ecological consequences of extinction also need to be considered. In the case of population replacement and substitution GDs, assessors have to place a greater emphasis on the exact heritable trait compared to GDs that cause removal of the organism from the environment.

One crucial aspect of GD is the cargo—the genetic elements that will be dispersed through the population. The recent case of hybridization and introgression of genetic elements from a released transgenic mosquito strain in Brazil (Evans et al., 2019) points to the key elements of ERA: What is the harm and how likely is this to occur? This particular case did not involve a gene drive, but it illustrates a point that would apply to gene drives. The genetic elements that were introgressed were from the transgenic mosquito genetic background, rather than an introduced transgene. Therefore, any particular effect that might be observed cannot be attributed to genetic engineering. This is an important paradigm for the internationally agreed comparative ERA approach.

A gene drive which is designed to kill an organism after reproduction would have a different likelihood to cause harm than one which prevents the organism transmitting disease.

In the past few years GD has been subject to regulatory consideration in the US^3^, Australia^4^ in Europe (BVL as office for the German Biosafety Commission “ZKBS”^5^ and by researchers (Oye et al., 2014; Akbari et al., 2015) in contained use facilities. To date, no government decisions on gene drive have been made for environmental releases. Nevertheless, the question of whether new environmental risk assessment (ERA) challenges are associated with GD organisms has also been addressed by scientific organizations (Redford et al., 2019) and researchers (Esvelt et al., 2014; Collins, 2018; Rode et al., 2019).

ERA utilizes a reasoned, structured approach to address uncertainty based on scientific and technical evidence (Wolt et al., 2010). Release of GD organisms into the environment currently has a high degree of uncertainty about how they would behave. Whilst modeling can help predict the outcomes (de Jong, 2017), additional data is required to determine if harm could occur during these kinds of releases. This additional information to improve risk assessment is data which is critical to assess risk to the environment (e.g., data on altered phenotype and population data rather than molecular data) (Layton et al., 2015).

Guidance on how to identify significant risks from organisms can be obtained from our experience with those organisms that cause harm. For example, there is a wealth of information on plants (including crops) that cause harm. These plants are generally known as weeds and weed scientists have well-developed methods to assess risks and harms from weeds (Pheloung, 2001; Standards Australia, 2006; Bourgeois et al., 2019). These methods have been successfully modified for use in the risk assessment of GM crops (Keese et al., 2014).

Ellstrand (2018) reviewed 14 well-documented situations where GMOs have been detected in wild or feral plant populations. These have occurred due to seed or pollen movement. Using the core principle of regulatory risk assessment “exposure” x “hazard” = “risk, gene flow (including GD) is the “exposure” component of the equation.” Despite gene flow occurring, to date an environmental “hazard” became apparent only in very few of the studied cases. The most significant of these is glyphosate-tolerant creeping bentgrass which has become a significant weed of irrigation canals in Oregon, USA (National Research Council, 2017). This weed can be controlled using other herbicides, but these chemicals may be less desirable, particularly near waterways (Beckie et al., 2004).

Similarly, for animals there is guidance on what harms pest animals cause in different environments from risk assessors who currently control pest animals (e.g., SA pest animal risk assessment guide). There is also guidance and vast experience from release of biocontrol agents to control invasive pests or pathogens in many countries of the world (Saunders et al., 2010; McColl et al., 2014), which would be applicable to gene drives.

Currently most GMOs are applied in the agricultural sector. GDs are different as most of the proposed applications are intended to modify wild populations. There are some proposed applications in plants (Neve, 2018), but generally. GD applications are seen as less relevant in plants or for use in agricultural systems (Duensing et al., 2018).

GD in wild animals providing fitness advantages for the hosting individuals will undoubtedly increase the environmental exposure of a GMO. It is thus very important to generate reliable data in the laboratory and from contained releases (e.g., islands) before the introduction into borderless/expansive environments. Nuclease-based GDs are the most advanced and thus the major focus: According to Redford et al. (2019) three types of information about the target and non-target species are required before implementing a gene drive strategy:

“*– Genetic and technical information needed include how to breed and conduct controlled experiments in the target species. Gene drive research also requires the availability of genome editing technology in the focal species or a related species, and the availability of an annotated reference genome to identify potential targets and design gRNAs that are specific of these loci (Moro et al.*, *2018**)*.

–* Ecological and evolutionary data on potential non-target species includes quantification of gene flow between target and non-target species (hybridization or horizontal gene transfer), checking for the presence of potential target sites in non-target species, and appropriate modeling of food web structure to forecast long-term ecosystem impacts (Moro et al.*, *2018**)*.

–* Ecological information needed includes behavioral and demographic data (e.g. spatio-temporal variation in size; Moro et al.*, *2018**), and a good understanding of the mating system and of gene flow between populations (e.g. quantifying dispersal ability as well as anthropogenic dispersal; Webber et al.*, *2015**). Spatially explicit theoretical models can help predict gene drive dynamics.”*

Two types of modeling are available supporting the ERA in the (inherent) light of uncertainty: population genetic models (e.g., de Jong, 2017) and spatial population models (e.g., Sánchez et al., 2019)

A threat for the sustainable use of GD systems is the development of resistance in target species. Prominent examples have already been identified in laboratory experiments (KaramiNejadRanjbar et al., 2018). Improved molecular designs may counteract rapid resistance evolution (Champer et al., 2019).

Whatever detailed guidance for GD will be developed in the future, it is important to take the lessons learned from the Cartagena Protocol Guidance on Risk Assessment of LMOs into account (Hokanson, 2019).

## Decision Making

Debates regarding the regulatory status of GD organisms generally follow a comparative approach with GMOs and with conventional organisms obtained by mutation or breeding. However, in case of GD organisms the likelihood and spread of genes into target and non-target populations is increased relative to comparators. Thus, it is the consequence of—successful—gene drive applications that needs to be finally assessed by decision makers.

As Redford et al. (2019) pointed out, *seeking to reduce epistemic uncertainty by performing a risk assessment on emerging technologies may require research activities that themselves pose some risk*. There will be tradeoffs between reducing uncertainty and avoiding risk that challenge the decision making process. Conducting field trials on isolated islands first and/or molecular confinement measures may be suitable steps forward.

Risk assessment and decision making for classical biological control for invasive species was reviewed by Teem et al. (in preparation), providing considerations involved in releasing an organism into the environment. The use of natural enemy species as biological control has been widely used and is accepted as an environmentally sound and effective means of reducing or mitigating the effects of pest species. Such natural enemies species have been successfully used to control invasive species all over the world.

Regulators and policymakers need to become familiar with the technical aspects of GD as well as the societal impacts. Of particular importance is public participation, stakeholder involvement and capacity building in “Release States.” Regulators need to contribute and encourage open and trustworthy GD research. A critical review of such “responsible” GD introduction is provided by Kuzma (2019) who argues that “external experts, stakeholders, and citizens with specialized and local knowledge” should be consulted in a more transparent way (Kuzma, 2019). This need for a change in communication style was also discussed by Brossard et al. (2019). Potential examples of how stakeholders are involved in specific projects can be found at the Target Malaria Project^6^.

## Harmonization of Regulation

In order to facilitate free global trade, internationally harmonized regulations are needed. However, it is not clear for GD whether this will be possible. It would require an international consensus for regulation which would require an organization to able to advance and coordinate this harmonization process. The questions around what is possible and who might advance this harmonization process will certainly be on the agenda of international conferences, e.g., under WHO or FAO leadership. One of the most crucial points here is the need for risk/benefit analysis in order to increase public awareness and also the awareness of the regulatory authorities and policy makers.

## Preparing for Future Gene Drive Applications

Risk and regulatory considerations for gene drive organisms will evolve considering the speed of introduction into the environment and the geographical location (Harvey-Samuel et al., 2019). At a workshop held at the Lorentz Center in Leiden 2017^7^, participants gave a rough forecast for the next 10 years:

Timeline of potential first environmental release of GD organisms:

- *Mus musculus* 2023
- Anopheles gambiae 2026
- *Felis catus* 2028
- *Rhinella marina* 2030.

## Conclusion

The increase in efficacy and decreasing costs will revolutionize the tools that science-driven economies will apply to fight against invasive species. These will include modern tools like GD. The right balance between precaution and innovation needs to be found for the benefit of society. The policy around regulation needs to balance the public's need for health, food, feed, and environmental safety with the economic costs for developers, growers, shippers and processers without wasting or damaging environmental resources. The importance of a globally harmonized regulatory approach is key to successful innovation. There is a general agreement that GD is a very powerful tool that needs careful and thorough evaluation before any release into the environment should be granted. It is still unclear whether a self-limiting GD is likely to be favored by regulators for approval compared to self-sustaining GD. Risk assessments for all gene drives will be on a case by case basis, so it is difficult to predict how different GD will be evaluated by risk assessors before they are assessed by regulators. Gene drive mouse and mosquitoes for invasive species control will be the likely test case for public acceptance of gene drive technology. A broad range of expertise, including ecologists, conservation geneticists, and nature reserve managers need to be involved. Responsible policy making benefits from engagement with stakeholders, policymakers, and local communities (Sirinathsinghji, 2019).

## Author Contributions

All authors listed have made a substantial, direct and intellectual contribution to the work, and approved it for publication.

### Conflict of Interest

The authors declare that the research was conducted in the absence of any commercial or financial relationships that could be construed as a potential conflict of interest.

## Footnotes

^1^The Cartagena Protocol on Biosafety to the Convention on Biological Diversity defines a so-called living modified organism (LMO) in Article 3 (g) as “any living organism that possesses a novel combination of genetic material obtained through the use of modern biotechnology.” Modern biotechnology is further defined in Article 3 (i) as “the application of: a. *In vitro* nucleic acid techniques, including recombinant deoxyribonucleic acid (DNA) and direct injection of nucleic acid into cells or organelles, or b. Fusion of cells beyond the taxonomic family, that overcome natural physiological reproductive or recombination barriers and that are not techniques used in traditional breeding and selection.”

^2^These amendments, arising from the Gene Technology Regulator's Technical Review of the Regulations (http://www.ogtr.gov.au/internet/ogtr/publishing.nsf/Content/reviewregulations-1), come into force 8 October 2019.

^3^https://www.aphis.usda.gov/aphis/ourfocus/biotechnology/am-i-regulated/Regulated_Article_Letters_of_Inquiry

^4^http://www.ogtr.gov.au/internet/ogtr/publishing.nsf/Content/53139D205A98A3B3CA257D4F00811F97/$File/Guidance%20on%20gene%20drives.pdf (accessed January 02, 2020)

^5^http://www.zkbs-online.de/ZKBS/EN/03_Fokusthemen/Gene%20Drive%20Systems/Gene%20Drive%20Systems_node.html (accessed December 30, 2019)

^6^https://targetmalaria.org/

^7^https://www.lorentzcenter.nl/lc/web/2017/872/info.php3?wsid=872&venue=Oort, accessed July 30, 2009.
